# Melatonin as a Therapy for Preterm Brain Injury: What Is the Evidence?

**DOI:** 10.3390/antiox12081630

**Published:** 2023-08-17

**Authors:** Silke Häusler, Nicola J. Robertson, Klervi Golhen, John van den Anker, Katie Tucker, Thomas K. Felder

**Affiliations:** 1 Division of Neonatology, Department of Pediatrics, Paracelsus Medical University Salzburg, 5020 Salzburg, Austria; 2 EGA Institute for Women’s Health, University College London, London WC1E 6HX, UK; n.robertson@ucl.ac.uk (N.J.R.); katie.tucker.17@ucl.ac.uk (K.T.); 3 Centre for Clinical Brain Sciences, University of Edinburgh, Edinburgh, 49 Little France Crescent, Edinburgh EH16 4SB, UK; 4 Pediatric Pharmacology and Pharmacometrics, University Children’s Hospital Basel (UKBB), University of Basel, 4001 Basel, Switzerland; klervi.golhen@ukbb.ch (K.G.); jvandena@childrensnational.org (J.v.d.A.); 5 Division of Clinical Pharmacology, Children’s National Hospital, Washington, DC 20001, USA; 6 Department of Laboratory Medicine, Paracelsus Medical University, 5020 Salzburg, Austria; t.felder@salk.at

**Keywords:** melatonin, preterm infants, inflammation, oxidative stress, oligodendrocyte dysmaturation, encephalopathy of prematurity, neuroprotection

## Abstract

Despite significant improvements in survival following preterm birth in recent years, the neurodevelopmental burden of prematurity, with its long-term cognitive and behavioral consequences, remains a significant challenge in neonatology. Neuroprotective treatment options to improve neurodevelopmental outcomes in preterm infants are therefore urgently needed. Alleviating inflammatory and oxidative stress (OS), melatonin might modify important triggers of preterm brain injury, a complex combination of destructive and developmental abnormalities termed encephalopathy of prematurity (EoP). Preliminary data also suggests that melatonin has a direct neurotrophic impact, emphasizing its therapeutic potential with a favorable safety profile in the preterm setting. The current review outlines the most important pathomechanisms underlying preterm brain injury and correlates them with melatonin’s neuroprotective potential, while underlining significant pharmacokinetic/pharmacodynamic uncertainties that need to be addressed in future studies.

## 1. Introduction

Following recent advances in neonatal medicine, the pathological spectrum of preterm brain injury has shifted from destructive brain lesions to primary cerebral dysmaturation disorders [1], where inflammation and oxidative stress (OS) play important roles in the initiation and maintenance of the pathogenic cascade resulting in preterm brain injury, known as encephalopathy of prematurity (EoP) [2,3]. In clinical practice, effective neuroprotective strategies are still lacking. Therapies that both mitigate the effects of prematurity and minimize the vulnerability of the preterm brain are required. Furthermore, treatment options should ideally be based on robust preclinical animal models that include the complex amalgam of disturbances encountered during the stepwise evolution of preterm brain injury—infectious and non-infectious inflammation—as well as an immature antioxidant defense system that predisposes to free radical damage [4,5].

Melatonin is a tryptophan-derived endogenous neurohormone that easily accesses the blood–brain barrier and functions as a free radical scavenger with anti-apoptotic and anti-inflammatory potential [6,7], making it a promising treatment option for preterm brain injury. Melatonin has shown promise in a variety of neonatal disorders (summarized and reviewed in [8]), but conclusive data on that topic is still lacking, necessitating well-designed future pre-clinical trials on the use of melatonin in the preterm setting. This review evaluates preterm infants’ susceptibility to inflammation as well as OS-induced pathology, with an emphasis on preterm brain injury. Additionally, it highlights the potential of melatonin as a valuable treatment option, given its pleiotropic beneficial effects as well as its favorable safety profile. Melatonin’s main biological and biochemical characteristics, as well as its pharmacokinetics and pharmacodynamics in the preterm preclinical setting, are discussed. Furthermore, the potential hazards and risks of melatonin treatment are reviewed, as is the need for continued research prior to its introduction into clinical practice.

## 2. Background: Pathophysiology of Preterm Brain Injury

Despite significant advances in neonatal care, there is still a clear link between preterm birth (PTB) and brain injury. Infection and/or non-infectious inflammation of the maternal/placental/fetal (MPF) triad occur in the majority of prematurely born neonates, sensitizing the developing brain to subsequent insults [9,10,11,12,13]. The immature brain passes through several essential sequences of CNS development during the last trimester of gestation: cell proliferation, differentiation, maturation (axonal outgrowth and dendritic arborization), migration, synapse formation, pruning, and an early phase of myelination that should provide for a well-orchestrated development of neural circuitry [14]. The speed and complexity of these processes account for their vulnerability to perturbations [9,15,16,17,18]. 

Preterm delivery is one of the major risk factors for preterm brain injury. Maintenance of pregnancy requires ongoing meticulously balanced fetomaternal immune adaptations from implantation until labor [11,19,20]. PTB, as demonstrated in preclinical animal models, represents a disruption in this immunological balance, causing an increase in the release of proinflammatory cytokines such as tumor necrosis factor (TNF)α, interleukin (IL)-1, IL-6, and IL-8 by maternal macrophages, which are major contributors to the induction of preterm labor, mainly following activation of the NF-κB pathway [21,22,23]. Proinflammatory mediators cross the placenta and enter the bloodstream, where they activate the fetal immune system [11,24,25]. Failure to resolve inflammation [26] results in dysregulated and prolonged inflammation, which can damage the immature organ system. This concept of a fetal inflammatory response syndrome (FIRS) with an abnormal fetal immune response and increased levels of proinflammatory cytokines (primarily IL-6) that are insufficiently balanced by immunosuppressive regulators (e.g., IL-10) [26,27,28,29] is regarded as a first inflammatory event sensitizing for subsequent pre- and postnatal “hits” [11,30]. In the absence of an infectious agent, an inflammatory cascade can be induced in the preterm setting by typical complications of prematurity such as respiratory distress syndrome (RDS), mechanical ventilation, patent ductus arteriosus (PDA), and other hemodynamic disturbances such as intraventricular hemorrhage (IVH) +/− posthemorrhagic ventricular dilatation (PHVD), all of which are accompanied by a prolonged elevation in proinflammatory cytokines [31,32,33,34]. 

Furthermore, sustained inflammation causes an increase in free radical production, altering normal redox balance and causing cellular OS [35]. Activated inflammatory cells release a significant amount of oxygen radicals, leading to tissue injury. In addition, cytokines released during sustained inflammation have been linked to changes in nitric oxide synthase (NOS) production patterns, further enhancing the OS environment [4,36,37]. Importantly, the rapid transition from the intrauterine low-oxygen environment to the air and often increased oxygen supply due to respiratory immaturity results in a hyperoxic environment, which, in addition to the status of prematurity per se, causes OS and mitochondrial damage [38]. Preterm infants are devoid of an effective antioxidant defense system (which they would have received in the third trimester of pregnancy) and thus have poor protection against OS. As a result, they are particularly vulnerable to the harmful effects of free radicals, culminating in the phenomenon of “oxygen radical disease in the (preterm) newborn” [4,36,37,38,39]. 

Moreover, preterm birth occurs frequently in pregnancies complicated by disorders such as preeclampsia, fetal growth restriction, maternal infections, and gestational diabetes, all of which are known to increase the oxidative burden [40]. Increased OS in this context overburdens the detoxification system of the developing brain’s preterm, immature mitochondria, resulting in bioenergetic dysfunction. Because mitochondria are responsible for cellular energy production in the preterm age, where high energy requirements are essential for postnatal organ development, mitochondrial dysfunction results in differentiation arrest and a failure in the maturation of the developing brain [5,36,41]. Sustained inflammation and free radical disease of prematurity, which are partially mutually dependent, may create a self-perpetuating cascade of peripheral inflammation and neuroinflammation via disruption of the blood–brain barrier. Once peripheral immune cells and cytokines enter the CNS, they can activate astrocytes and microglia, causing them to adopt a proinflammatory phenotype and thus propagate the central inflammatory response [10,11,27], culminating in the multifaceted EoP [3,10,11,17,42].

### 2.1. Vulnerability of the Developing Preterm Brain to Disruption and Injury

The decreasing prevalence of previously observed necrosis and associated axonopathy (though still relevant in severe insults) [9,18] has increased interest in other mechanisms responsible for the only modest improvement in neurodevelopmental disabilities in extremely preterm infants [43]. In preterm neonates, especially those of extremely low gestational age, the underlying pathology is a combination of cerebral diffuse white matter injury (WMI) followed by a prolonged period of dysmaturation in both white and grey matter structures [15,16,17,18,44]. Apart from hypoxic-ischemic events that occur before, during, or shortly after birth, microglia activation and reactive astrogliosis play important pathophysiological roles in this context [17,45,46,47,48]. During this developmental stage (23–32 weeks postconceptional age, where premature birth often occurs), GABAergic interneurons, oligodendrocyte precursor cells (OPCs), and pre-oligodendrocytes (pre-OLs) are particularly vulnerable cells. Indeed, pre-OLs are thought to be the most important cellular targets in preterm brain damage. Their maturation-dependent sensitivity is supposed to arise from an abundance of excitatory receptors and an immature antioxidant system combined with mitochondrial dysfunction [9,15,16,18,49,50]. A disruption of oligodendrocyte-axonal integrity with poor trophic supply in the event of OL dysmaturation is of concern for axonal growth, function and survival, which may have a deleterious influence on the neuronal connection in different parts of the brain via decreased synaptogenesis [1,15,16,42].

### 2.2. The Need to Develop Treatment Strategies to Ameliorate Sequelae of Preterm Brain Injury

There are currently limited treatment options available to improve outcomes following preterm brain injury. Interventions may be “neuroprotective”, i.e., preventing the initial (inflammatory) insult, or “neurorestorative”, i.e., targeting subsequent brain maturation disturbances (i.e., dysmaturation, hypomyelination and impaired neural circuitry) [51]. Overall, the ideal treatment would be capable of combating various noxious insults that comprise the multiple-hit pathology [26] in the progression of EoP. Melatonin, which has a broad range of potential as a neuroprotective and neurorestorative agent [7,8,52,53,54,55,56,57], seems highly promising in this context. 

## 3. Melatonin—Synthesis and Function

### 3.1. Biological and Biochemical Aspects

Melatonin (*N*-acetyl-5-methoxytryptamine) is an endogenous neurohormone that is synthesized from L-tryptophan via a series of enzymatic steps prior to secretion [58]. The release of melatonin is controlled by hydroxyindole-O-methyltransferase, which is regulated by the photoneural system and thereby follows a circadian rhythm [59]. In humans, it is mostly produced by the pineal gland, with minor contributions from the retina, lymphocytes, bone marrow, gastrointestinal system, and thymus [60]. Melatonin production (and metabolization) in mitochondria is important in this context since mitochondrial melatonin levels are significantly greater than plasma levels [61,62]. Melatonin is primarily protein bound in the circulation and, due to its lipophilic nature, can easily cross biological membranes to reach cells and tissues in other compartments. 

**Figure 1:**
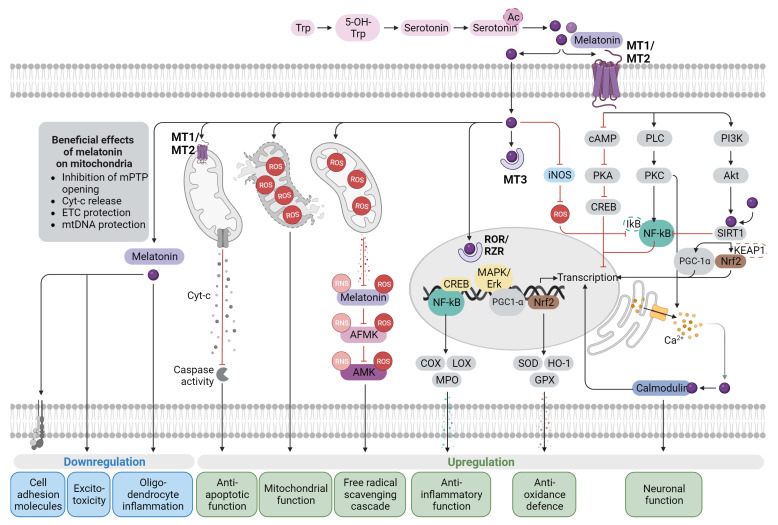
Melatonin signaling mechanisms: Melatonin shows receptor-mediated and receptor-independent activity and interacts with a variety of cellular signaling pathways. Both MT1 and MT2 are members of the GPCR receptor family. Melatonin binding and signaling involve several effectors, including PKC, PLC, and PKA, as well as downstream signaling pathways. Melatonin can also enter the cell through passive diffusion and transporters, where it activates mitochondrial receptors or cytosolic quinone reductase 2 (MT3), which in turn activates nuclear receptors ROR/RZR. Melatonin causes a shift towards an anti-inflammatory and antioxidant response and the preservation of mitochondrial and neuronal integrity. (Abbreviations: 5-OH-Trp, 5-Hydroxytryptophan; AMK, *N*^1^-acetyl-5-methoxykynuramine; AFMK, *N*^1^-acetyl-*N*^2^-formyl-5-methoxykynuramine; Akt, protein kinase B; COX, cyclooxygenase; CREB, cAMP response element-binding protein; Cyt, cytochrome c; ETC, electron transport chain; GPX, glutathione peroxidase; HO, heme oxygenase; IκB, Inhibitory-kappa B; iNOS, inducible nitric oxide synthase; KEAP1, Kelch-like ECH-associated protein 1; LOX, lipoxygenase; MAPK, mitogen-activated protein kinase; MPO, myeloperoxidase; mPTP, mitochondrial permeability transition pore; MT1/2/3, melatonin receptors 1/2/3; mtDNA, mitochondrial DNA; NF-κB, nuclear factor- kappa B; Nrf2, nuclear factor erythroid 2-related factor 2; PI3K, phosphoinositide 3-kinase; PKA, protein kinase A; PGC-1α, peroxisome proliferator-activated receptor gamma coactivator 1-alpha; PKC, protein kinase C; PLC, phospholipase C; RNS, reactive nitrogen species; ROR/RZR, retinoid acid receptor-related orphan receptor/retinoid Z receptor (RZR); ROS, reactive oxygen species SIRT1, sirtuin-1; SOD, superoxide dismutase; Trp, tryptophan).

Melatonin regulates several physiological processes and exerts its effects via both cellular receptors and receptor-independent mechanisms that are interconnected (Figure 1). Receptors mediating the action of melatonin include the G protein-coupled receptors MT1 and MT2, the cytosolic MT3 receptor (also called quinone reductase QR2), and the nuclear receptors ROR/RZR [63,64,65]. Melatonin receptors (MT1, MT2, and MT3) are variably expressed on target organs and are primarily responsible for endocrine, autocrine, and paracrine effects [66]. MT1 is abundant in the suprachiasmatic nucleus, the central pacemaker of the hypothalamic circadian timing system. Furthermore, MT1 is expressed in the substantia nigra, cerebellum, nucleus accumbens and cortex [67], as well as in peripheral tissues outside the CNS. MT2 is less widely distributed and is found in the retina, hippocampus, cortex, and cerebellum [68,69]. Furthermore, both MT1 and MT2 expression, as well as nuclear receptors, have been identified in human immune cells [70,71,72]. 

Melatonin catabolism occurs via enzymatic or non-enzymatic pathways. Melatonin is degraded by the indolic pathway via hepatic CYP450-mediated hydroxylation, followed by sulfation or glucuronidation prior to urinary excretion. The bioactive metabolite *N*^1^-acetyl-*N*^2^-formyl-5-methoxykynuramine (AFMK) can be generated by the independent kynuric pathway. AFMK is formed enzymatically or through non-enzymatic reactions with free radicals or ultraviolet B radiation. Melatonin can also be demethylated to *N*-acetylserotonin by CYP2C19 or CYP1A [73]. Non-enzymatic pathways include the formation and reaction of melatonin radical species with reactive oxygen species, reactive nitrogen species, oxoferryl hemoglobin, or hemin. As a result, in addition to melatonin, its precursors and metabolites are effective in the detoxification of free radicals, extending the time of action [74,75,76].

### 3.2. What Preterm Infants Lack in Terms of Endogenous Melatonin Synthesis and Circadian Secretion during (Late) Pregnancy

The fetus does not secrete melatonin throughout the prenatal period and is therefore dependent on maternal melatonin secretion via transplacental transfer [77]. This is because the pineal gland does not become functionally active until after birth. For the first 2–3 months after full-time birth, the initiation of pineal melatonin secretion seems to be impaired [78,79,80,81]. Nonetheless, term newborns may be provided with and trained in circadian rhythmicity by their mother’s own milk, which contains circadian fluctuations in melatonin concentrations (during breastfeeding) [82]. Preterm neonates appear to have an even more prolonged period of melatonin deprivation because the neurological circuitry that controls melatonin production is also immature [79,83]. When infants are subjected to fetal distress or fetal growth restriction, the initiation of pineal secretion appears even more delayed [84]. Furthermore, preterms may lack chronobiological adherence and rhythmicity throughout their NICU stay due to a dyssynchronized feeding schedule (because of a mismatch between the time of expression and the time of feeding) [82].

Circadian melatonin secretion rises progressively during the third trimester, peaking at roughly 36 weeks postconceptual age, most likely due to de novo melatonin placental synthesis [85,86]. Melatonin crosses the placenta from the maternal circulation to the fetus, where melatonin receptors are abundantly expressed in both central and peripheral tissue [87,88]. The connection between the circadian clock system and the maternal immune system is crucial for the maintenance of pregnancy as well as for preventing other pregnancy-related inflammatory disorders, such as gestational diabetes and preeclampsia [88]. The integrity of circadian rhythmicity promoted by melatonin secretion is also critical for appropriate fetal neurodevelopment; this may occur primarily via favoring the REM sleep states, known to be important for neurogenesis [89]. Given that the human brain experiences a significant growth spurt during the third trimester of pregnancy and during the neonatal period, chronodisruption caused by maternal melatonin deprivation in the preterm infant may have negative consequences during a critical period of brain development with limited anti-inflammatory defense mechanisms [90,91].

## 4. Preclinical Data Supporting the Use of Melatonin

### 4.1. Preclinical Models of Preterm Brain Injury

The effects of prenatal and postnatal melatonin treatment have been studied in preclinical models of preterm brain injury. There is a consistent and compelling reduction in brain injury with melatonin across multiple animal models of preterm brain injury, as previously summarized [92] and confirmed in subsequent studies within the last decade (Table 1).

#### 4.1.1. Antenatal Treatment

Prenatal effects of melatonin have been well studied [93,94,95,103]: melatonin applied to the mother readily crosses the placenta and reaches fetal circulation with virtually no toxic effects on fetal development, as demonstrated in dose escalation studies using markedly supraphysiological doses [104,105]. Aside from its potential in preventing pregnancy-associated maternal complications (i.e., GMD and preeclampsia), which is beyond the scope of this review, cumulative data suggests that maternal melatonin treatment has neuroprotective effects in the fetus, reduces preterm birth, and promotes fetal survival [94,106,107]. 

In mouse models of lipopolysaccharide (LPS)-induced inflammatory brain damage, prophylactic antenatal melatonin treatment has been shown to have neuroprotective effects via decreased microglial/macrophage activation, decreased production of pro-inflammatory mediators, decreased expression of NOS isoforms, and prevention of epigenetic modifications of normal fetal brain development [93,94,108]. In a sheep model with intrauterine growth restriction and consequently chronic hypoxia, daily maternal antenatal melatonin administration until the end of labor resulted in significant improvements in neurological function (neonatal behavioral assessment). Furthermore, when compared to sheep with sham infusions, histopathological examination of the brain revealed significantly less OS-induced damage as well as a normalized myelination pattern [103].

#### 4.1.2. Postnatal Treatment

- *Protection against Excitotoxicity*

Excitotoxicity, which is triggered by episodes of inflammation as well as hypoxia/ischemia (HI), plays an important role in CNS injury in preterm infants—the accumulation of extracellular glutamate due to increased release and impaired uptake injures the fragile immature oligondendrocytes and neurons [109,110]. Injections of glutamate agonists into various areas of the brain in different animal models have revealed patterns of perinatal brain injury comparable to those seen in human preterm infants (reviewed in [110]). After intracerebral injection of the glutamate analog ibotenate in 5-day-old (P5) mouse pups, melatonin’s neuroprotective effect was based on both an early effect by preventing blood–brain barrier (BBB) disruption and a late effect by promoting white matter lesion repair and inducing axonal regrowth and sprouting [111,112]. The rapid restoration of BBB integrity was assumed to be achieved through modification of tight junction protein expression, an effect that was probably not MT1 and MT2 dependent but could have an impact further down in the signal transduction cascade [112]. In fact, in a subsequent rodent pup model of HI injury, the inhibitory effect of melatonin administration on BBB permeability disruption was related to the inhibition of the microglial toll-like receptor 4/nuclear factor-kappa B (TLR-4/NF-κB) signaling pathway [100]. In contrast, the neuroregenerative effect, i.e., the stimulation of secondary white matter lesion repair, is assumed to be MT-dependent, as luzindole, a selective MT antagonist, inhibited the effects of melatonin in a previous study [111]. Importantly, Husson et al. [111] demonstrated dose-dependent as well as region- and cell-specific efficiency on excitotoxic lesions and cell death in the brain with a single intraperitoneal (i.p.) melatonin administration (0.0005 to 5 mg/kg) within 4 h after the injury. Melatonin (given i.p. at a dose of 5 mg/kg immediately before the noxious agent) was shown to preserve the ability to acquire associative abilities and to ameliorate learning disabilities in brain-lesioned P5 mice receiving intracerebral ibotenate [113].

- *Protection against Oxidative Stress and apoptosis*

Melatonin acts directly as a potent free-radical scavenger via its non-receptor-mediated action and indirectly by modulating the antioxidant defense system by promoting the synthesis of antioxidant enzymes and stabilizing the mitochondrial electron transport chain activity to reduce ROS leakage. As a result, a highly effective sequential interaction between free radicals and melatonin and its metabolites is ensured, and a potent antioxidant cascade is formed [114]. Melatonin’s antioxidant activity in neonatal encephalopathy has been abundantly demonstrated in numerous small and large animal studies, with decreased amounts of OS markers, reduced inflammation, and attenuated cell death (as already summarized elsewhere [54,92]). Melatonin reduces oxidative damage to cerebral lipids, improves secondary cerebral energy failure, increases glutathione (GSH) in periventricular white matter, and so inhibits neuronal apoptosis and subsequent neurological impairment after free radical noxious events [95,115,116,117]. 

Melatonin’s protective properties in the OS scenario have been demonstrated in HI animal models as well as after endotoxic LPS stimulation. HI was induced in two fetal sheep models by complete umbilical cord occlusion at 0.7 gestation [96,97]. In the study by Drury et al. [96], melatonin infusion (0.1 mg/kg bolus followed by 0.1 mg/kg per hour over 6 h) was started immediately before the occlusion and subsequently given once a day for 6 days afterwards. Sheep were euthanized 7 days after recovery in utero. Yawno et al. [97] started i.v. melatonin postnatally 2 h after the HI insult (at a bolus dose of 0.2 mg followed by 0.1 mg/h for 24 h) to better mimic the clinical situation when treatment may not begin immediately after birth. Sheep were euthanized and studied 10 days after the insult. In both studies, melatonin demonstrated potent antioxidant properties and was able to decrease OS, apoptosis, inflammation and microglia activation within the white matter. Melatonin also demonstrated intermediate, region-specific protective benefits on developing white matter, with partial restoration of mature oligodendrocytes and restored myelin morphology in certain parts of the brain. This incomplete effect was assumed to be related to regional differences in blood flow, which melatonin has been shown to influence as well [118]. Furthermore, as Drury et al. observed, melatonin-treated sheep showed significantly faster recovery of fetal EEG. 

Melatonin was reported to significantly attenuate OS-induced neurobehavioral abnormalities and brain damage in a rodent model after LPS exposure [98]. Melatonin (20 mg/kg) significantly reduced nitrosative and oxidative stress, as well as the amount of inducible nitric oxide synthase (iNOS)+ cells, after an i.p. injection of LPS on P5 animals. Furthermore, melatonin significantly reduced the LPS-induced proinflammatory cytokine response as well as the number of activated microglia in the neonatal rat brain. A subsequent study on near-term rodents provided possible mechanisms of melatonin action in neuroprotection against LPS-induced OS, acute neuroinflammation, and neurodegeneration. The authors demonstrated that melatonin administration to P7 rats stimulated the sirtuin-1/nuclear factor erythroid 2-related factor 2 (SIRT1/Nrf2) signaling pathway, thereby attenuating excess ROS production and the ensuing NF-κB activation, resulting in decreased iNOS and COX-2 formation. Melatonin also completely blocked LPS-induced glial cell activation, including both microglia and astrocytes, as well as the associated acute neuroinflammation. Thus, two putative mechanisms were identified in this work to explain how melatonin could inhibit the developing cascade from OS to accumulate inflammation and spread neurotoxicity [119].

Melatonin’s antioxidant and free radical scavenger effects rely significantly on mitochondria to maintain brain tissue integrity. Melatonin’s protective activities against mitochondrial dysfunction culminating in neuronal (apoptotic) cell death have been reviewed in detail [8,120]. In preclinical animal models following acute perinatal hypoxic events, melatonin treatment was clearly shown to reduce apoptotic neuronal loss (e.g., [95,97]).

In an adult rodent model, following middle cerebral artery occlusion, melatonin’s antiapoptotic effects were reported to be MT1/2 receptor-mediated [121]. Remarkably, in this context, Suofu et al. demonstrated in an elegant study on mice that mitochondria, in addition to synthesizing and releasing melatonin, express a selective G protein-coupled MT1 receptor in their outer membrane. Based on their findings, they postulated the existence of an “automitocrine” signaling pathway by which mitochondrial melatonin may be able to mitigate stress-induced mitochondrial cytochrome c release and downstream caspase activation in an autocrine fashion [122]. It is tempting to speculate whether this neuroprotective method is also relevant in the preterm brain, but this remains elusive.

- *Protection against neuroinflammation*

Melatonin is a potent immunomodulator with a significant impact on the immune system under different pathologic conditions. Melatonin’s anti-inflammatory properties have been well documented [8,123,124,125,126]. Remarkably, depending on the stage of inflammation, the action of melatonin may be more complex. It also appears to have a transitory pro-inflammatory effect during the early phases of inflammation. However, anti-inflammatory effects may predominate in the context of chronic inflammation, which may be more relevant to the preterm intrauterine and extrauterine milieu [127,128,129]. 

In a rodent model, LPS was injected i.p. during late pregnancy, imitating and attempting to investigate the effects of chorioamnionitis on the developing brain. In this experimental setting, pregnant rats were given LPS and melatonin (5 mg/kg) during embryonic days 19 and 20 (E19 and 20). Melatonin, by reducing pro-inflammatory gene expression and microglial activation in the neonatal brain, was able to attenuate LPS-induced sensitization to a second excitotoxic shock (ibotenate) on postnatal day 4. Melatonin completely blocked the LPS-induced inflammatory cascade by reversing the effects of LPS on endoplasmic reticulum stress proteins and autophagy proteins. Furthermore, SIRT1 expression was preserved, and deregulation of specific microRNAs was prevented. As a result, in the melatonin-treated pups, the second hit (ibotenate)-induced brain lesion size was reduced by 40% compared to animals devoid of melatonin [99]. 

Following infection and OS, significant attention has been focused on the activation of nucleotide-binding domain-like receptor protein 3 (NLRP3) inflammasomes, a multiprotein platform that maintains and even aggravates the harmful inflammatory cascade [102,130]. An interesting article reported on the effect of melatonin on the NLPR3 inflammasome after three noxious events. Following an inflammatory (LPS injection), ischemic (ligation of the right carotid artery), and hypoxic (90-min stay in a chamber filled with 8% O_2_ and 92% N_2_ standard gas) insult to neonatal P3 rats, the animals were euthanized and studied on P7. Melatonin (15 mg/kg) was given to the treatment group before the noxious insults and once daily thereafter. Melatonin was shown to inhibit the increased activity of NLRP3 inflammasomes by both enhancing mitochondrial autophagy and inhibiting TLR4/NF-κB pathway activity. As a result, melatonin significantly attenuated pathological changes in white matter disease when compared to the control group [102].

The relevance of microglia in neuroinflammation is widely accepted. Melatonin reduces microglial inflammation by creating an anti-inflammatory interaction network via a variety of mechanisms, as reviewed in detail by Gao et al. [131]. The resident brain immune cell polarization is important in the transduction of peripheral neuroinflammation [132]. Recent research suggests that neuroinflammation may induce reactive microglia to polarize into M1 (a classically activated, proinflammatory microglial phenotype) and M2 (an alternatively activated, anti-inflammatory microglial phenotype) [133]. In a P1 rodent model, LPS was i.p. injected, followed by i.p. administration of melatonin (10 mg/kg). Melatonin was given once daily until P7 in this experimental model. In melatonin-treated rat pups, microglia polarized towards an M2-anti-inflammatory phenotype, whereas in LPS-treated pups, microglia polarized towards an M1-proinflammatory phenotype. Furthermore, the effect of melatonin on polarizing microglia from the M1 to M2 phenotype was blocked by luzindole, a melatonin receptor inhibitor, indicating that the change from the M1 to M2 phenotype may be receptor-dependent. The authors were able to demonstrate that the JAK2/STAT3/telomerase pathway was involved in the melatonin-induced polarization of microglia [101]. 

Favrais et al. reported on the partial protective effects of melatonin according to the cell type and region investigated in another rodent model, examining the effects of chorioamnionitis at different time points during postnatal brain development [134]. Following the prenatal i.p. injection of LPS into pregnant rats, melatonin (5 mg/kg) was applied once by the same route immediately thereafter. Melatonin was shown to prevent a regional decrease in GABAergic neurons (an important cell type during brain development, orchestrating the ordered formation of neuronal circuits at the structural and functional levels). However, it failed to significantly impede the LPS-induced alterations in OL maturation, despite its dampening effect on systemic and brain inflammatory responses (determined by measuring proinflammatory cytokines as well as cleaved-caspase-3-positive cells). The authors suggested that the timing of exposure in relation to the cell maturation stage, as well as the timing of melatonin application, might be crucial in this context.

- *Promoting OL maturation and myelination*

Several studies on the protective effect of melatonin on WMI have focused on its ability to reduce (white matter) inflammation by inhibiting microglial and reactive astrocyte activation or to induce polarization of these cells toward an anti-inflammatory phenotype, restoring normal OL maturation (see above). Melatonin receptors MT1 and MT2 are expressed not only by microglia and astrocytes but also by oligodendrocytes [55]. The effect of melatonin at different doses was investigated in a preclinical preterm rodent model following unilateral carotid artery ligation on E17. In the experiments performed, the authors could observe a direct, maximal effect of melatonin at a dose of 0.2 mg/kg, administered from P1 to P3, on oligodendroglial maturation and myelination [55]. A similar finding was achieved by Wong et al., who found that melatonin injected into P5 rat pups successfully prevented pre-OL decrease after LPS stimulation [98]. In long-term follow-up, however, Favrais et al. found no difference in OL maturation and myelination favoring the melatonin group [134]. In line with this observation, Drury et al. and Yawno et al. reported an incomplete restoration of immature OLs with melatonin administration in HI preterm sheep models [96,97].

Only recently was it shown that melatonin might have neurotrophic potential [135,136,137], which adds to the interest in its effects on oligodendrogenesis. Melatonin was reported to increase the percentage of MBP+ cells in embryonic mouse-derived NSC cell cultures [138]. Similar increases in OL numbers resulted from melatonin administration to NSC neurospheres cultured from the adult mouse [139].

As yet, the results are ambiguous, and the precise mechanism(s) involved in melatonin’s maturation effect on developing oligodendrocytes and the subsequent myelination process—as well as the timing and duration of treatment—remain elusive and a basis for future research.

- *Tertiary phase of brain injury—neurorestorative potential following delayed melatonin administration in combination with erythropoietin*

There is increasing evidence that a tertiary phase of brain injury may play a role in the formation and extent of clinical manifestations of EoP. This phase may last for months (or perhaps years), allowing for a window of therapeutic opportunity [140]. After prolonged complete umbilical cord occlusion, chronically instrumented preterm fetal sheep were studied recently to simulate the different phases of brain injury. In histological examinations at different time points following the insult, the evolution of cystic white matter lesions and degeneration was closely followed. Severe white matter degeneration, including white matter atrophy, ventriculomegaly, and overt cystic white matter lesions, was present only on day 21 of evaluation but not at the earlier investigated time points, namely 3, 7 and 14 days. Based on these findings, the authors speculated that there would be a relatively long therapeutic window for treating cystic white matter injury [141].

In addition, in a rat model of preterm birth following dual intrauterine injuries from placental malperfusion and chorioamnionitis on E18, melatonin, in combination with erythropoietin (EPO), dampened molecular, sensorimotor, cognitive, and social abnormalities in adult rats. In this model, a 10-day’ course of melatonin (20 mg/kg) and EPO (2000 U/kg), beginning on P1, was applied, along with highly translatable testing platforms similar to those used in humans. The combination therapy normalized behavior, social interaction, and executive function, as well as hindlimb deficits [57].

In a subsequent study from the same group, using the same rodent model but starting melatonin at P15 (equivalent to a human infant one year of age) and continuing once daily until P20, melatonin normalized multiple gait metrics investigated, even in mature/adult animals, indicating an extended neuroreparative effect of this treatment strategy [56]. The underlying cellular and molecular processes behind the neuroregenerative effects of combined EPO + melatonin regimens remain unknown. In a landmark paper, Lin et al. demonstrated that children born very preterm who subsequently develop cerebral palsy (CP) symptoms have a persistent hyper-reactive immune system profile during childhood [142]. As recently demonstrated [143], ongoing inflammation may have a deleterious impact on endogenous neurorestorative processes in the CNS. Because melatonin and EPO are pleiotropic regulators of both the growing immune and neurological systems, the authors speculated that they could be promising candidates for neuro-immunomodulation and neuroregeneration, respectively.

## 5. From Bench to Bedside—Translation into Clinical Use

### 5.1. Pharmacological Use of Melatonin Pharmacokinetics

Melatonin’s pharmacokinetic (PK) profile in adults has been well documented. Secretion in healthy young adults follows a circadian rhythm, with average daytime and peak nighttime values ranging from 10 to 60 pg/mL, respectively [81,144]. Preterm infants, particularly neonates <34 weeks postconceptual age, are melatonin deficient (in the majority of cases < 7 pg/mL), with concentrations being consistently lower even at term equivalent age when compared to infants born full term [83]. There is also no diurnal variation following premature birth [79,83]. There are multiple challenges to collecting good PK data on melatonin in preterm newborns. Preterms have a significantly lower body fat content than adults, which affects the distribution volume of lipophilic substances like melatonin. In addition, concerns about the degree of absorption probably complicate proper dosing and dosage regimen finding. Co-administration of frequently used drugs such as caffeine, which may enhance melatonin concentrations in the blood by competing for the same metabolic pathway [145,146], adds to interindividual variability in drug disposition in the preterm population [147,148]. 

Moreover, immature liver metabolism and poor renal excretion in neonates (probably even more pronounced in preterm infants) may also significantly impact the PK profile of melatonin. While the elimination half-life (t_1/2,z_) of melatonin in adults is 40–60 min [149,150,151], irrespective of the route of administration, in the preterm population, t_1/2,z_ is reported to be significantly prolonged in previous PK studies. We are aware of only two PK studies performed on preterm infants: Merchant et al. studied the melatonin intravenous dosage range in neonates <31 weeks of gestation. Results suggested that a melatonin concentration of 0.1 mg kg^−1^ h^−1^ infused over 2 h increased blood melatonin concentration to approximately the physiological peak adult values. The melatonin elimination half-life in this study was 15 h [145]. 

Another study investigated the pharmacokinetic profile of oral (intragastric) melatonin in preterm neonates aged 26 to 36 weeks and found that a single intragastric dose resulted in high peak plasma concentrations and area under the curve (AUC) values, indicating optimal oral absorption, even though the reported bioavailability of oral melatonin was only 3% in adults [149]. In this study, melatonin elimination half-life ranged between 7 and 11 h; this is similar to the results following intravenous infusion from Merchant et al. and higher than the adult values [145]. Another important finding of this study was the detection of a steady-state residual concentration after three doses of melatonin. The reported high peak plasma concentrations and prolonged half-life after melatonin administration in preterm infants suggest that a single dose of melatonin repeated every 12 to 24 h may be appropriate [152]. Thus, several uncertainties in PK characteristics in preterm infants make the identification of a safe and potentially effective dose of melatonin as well as the route of administration challenging, even more so when applied repeatedly and when considering the extent of immaturity/prematurity.

### 5.2. Term Brain Injury Models and Clinical Translation

Neonatal encephalopathy (NE) is a heterogeneous, clinically defined syndrome characterized by disturbed neurologic function in the earliest days of life in an infant born at or beyond 35 weeks of gestation, manifested by a reduced level of consciousness or seizures, often accompanied by difficulty initiating and maintaining respiration, and by depression of tone and reflexes. Despite treatment with therapeutic hypothermia (HT) in developed countries, only 60% of babies with neonatal encephalopathy (NE) at term or near term survive without cerebral palsy or neurocognitive impairment, with important societal consequences. Novel adjunct therapies are needed. 

In several preclinical models (rodent, lamb, and piglet), melatonin has been shown to be safe with robust brain protection at pharmacological levels as a single agent and combined with HT; the standardized mean difference (SMD) of combined outcomes (infarct size, neurobehavioral outcome, cell death) on meta-analysis is −0.92, 95% CI (−1.26 to −0.58). In vitro and efficacy studies suggest a concentration-dependent reduction in cell death with melatonin and a need for early dosing. Melatonin with an ethanol excipient is more beneficial than non-ethanol formulations (SMD −1.14, 95% CI (−1.64 to −0.65)) [153]. 

Over the last 10–15 years, the UCL [116,154,155,156] and Monash groups [115,157] studied melatonin in neonatal piglet and newborn lamb HI models, respectively, showing robust protection with and without HT, particularly using melatonin in an ethanol (2.5%) excipient. In vitro and in vivo piglet studies suggest that the optimal melatonin target level for optimal protection is 15–30 mg/L, achieved within 3–6 h of birth. As the oral bioavailability of melatonin is variable in sick newborn babies with NE, an intravenous melatonin formulation is needed to attain therapeutic concentrations as soon as possible after birth. A phase I dose escalation safety study is starting in 2023 with an intravenous formulation of melatonin in babies with moderate–severe NE who are given therapeutic hypothermia (ACUMEN Study—Acute High Dose Melatonin for Encephalopathy of the Newborn). However, the therapeutic level for protection after an acute HI insult may be different from that needed for the low-grade chronic inflammatory and oxidative stress seen over weeks in the preterm infant undergoing intensive care in the NICU.

### 5.3. Preterm Brain Injury Models and Clinical Translation

The therapeutic dosage of melatonin and the duration needed for protection in the preterm newborn are unknown. However, unlike traditional anti-inflammatory drugs that may exhibit severe adverse reactions during long-term administration, melatonin can be administered without serious side effects, making it a very attractive treatment option even in preterm infants [92]. Melatonin therapy has been shown to improve respiratory disorders such as RDS and BPD in preterm infants during the perinatal period [158,159]. 

Data on its efficacy in preventing/restoring preterm brain injury in the clinical context is limited. In the MINT study, the neuroprotective effect of intravenous melatonin (given once a day for 7 days starting 48 h after birth at a dose of 0.1 mcg/kg/h for 2 h) was evaluated in 58 preterm infants born at less than 31 weeks gestation. This study aimed to increase melatonin to physiological adult levels and not supra-physiological or pharmacological levels, which may be required to combat chronic inflammatory and oxidative stress in infants born very preterm. No significant difference in white matter fractional anisotropy as measured in MRI was detected [160]. The PREMELIP study (ClinicalTrials.gov Identifier: NCT02395783), which used MRI to assess the neuroprotective effect of melatonin administered in the immediate prepartum period in very preterm infants (<28 weeks gestation), was terminated without any given reason. A randomized controlled trial on melatonin has recently initiated recruitment to investigate its potential to prevent brain impairment following preterm birth. Melatonin is administered orally after birth at a dose of 3 mg/kg/day for 15 days, starting on postnatal day one, to infants born <30 weeks. Measures of clinical and radiological outcome, as well as neurodevelopmental testing, will be used. The results are pending (ClinicalTrials.gov Identifier: NCT04235673). The “Protect Me Trial” is a randomized controlled clinical trial assessing antenatal maternal melatonin supplementation (30 mg per day, until birth) for fetal neuroprotection in early-onset FGR (between 23 + 0 and 31 + 6 weeks gestation). Neurodevelopmental outcomes at 2 years of age are assessed in this study. Neonatal and infant neurodevelopmental testing will be performed, and children will be followed up until 2.5 years of corrected age. So far, no results have been published (ClinicalTrials.gov Identifier: NCT05651347).

## 6. Melatonin Treatment: Risks and Hazards—Current and Future Demands for Ongoing Research

Despite continuing research on this topic, preterm birth remains associated with a high burden of chronic neurodevelopmental impairment. The “ideal” animal model that reflects and combines all the pathologies underlying the development of EoP remains elusive. Preclinical models of brain injury have mostly focused on one aspect of the multifactorial pathology of EoP. However, combining multiple hits and their different effects depending on the time they occur—either before, during, or after birth—in a rapidly developing system, even if practically impossible to simulate, is paramount for the best-fit combination of interventions to ameliorate individual preterm neurodevelopmental trajectory. 

Furthermore, patient stratification based on interindividual risk profiles according to gestational age, sex, disease onset and progression would be desirable. In this context, reproducible biomarkers for identifying high-risk preterm infants as well as tracking disease progression would be extremely useful in better characterizing which neonates benefit from therapy initiation and clearly may contribute to a better understanding of the therapeutic window of opportunity. Although preventing the initiation of inflammatory cascades is important, a caveat in developing neuroprotective treatment strategies is a thorough understanding of the precisely timed and developmentally well-organized balance between maintaining the normal physiological role of inflammation (e.g., pruning) and reducing the pathological inflammatory response. Any clinically useful therapy must therefore avoid disrupting the physiological role of inflammation in normal brain development. As a result, the development of clinically effective neuroprotective anti-inflammatory agents requires coordinated preclinical studies that systematically address this issue. Above that, preterm brain injury, in its complexity, has been shown to be a dynamic disease with continuing inflammation and altered epigenetics, that predisposes increased cognitive impairment and sensitization to further insults [161]. This tertiary phase of brain injury, on the other hand, offers a therapeutic window for neuroregenerative approaches that may be applied to an already disrupted brain. Melatonin’s neuroprotective and neuroregenerative potential in preclinical studies virtually predisposes it to clinical use in the preterm setting, administered as a prophylactic or post-insult means of treatment, either as a single agent or in combination (e.g., with EPO) to further augment the neuroreparative effect of either treatment alone. 

However, there is a lack of data on melatonin dosing in human neonates. Most research has been dedicated to evaluating the effects of melatonin following hypoxic-ischemic encephalopathy (HIE) in term infants. While the preclinical data is compelling, clinical studies published thus far have primarily been observational trials, with only a few randomized controlled trials (RCT) including small numbers of participants without long-term follow-up data [162]. This lack of information is even more pronounced in the case of melatonin treatment in preterm infants. Several experimental studies have revealed that (antenatal and postnatal) melatonin supplementation has significant neuroprotective potential in various animal models of brain lesions that resemble lesions observed in human preterm neonates in several single models of brain injury [92]. Nonetheless, translation into clinical practice remains problematic due to uncertainties regarding optimal dose, time intervals, route and frequency of administration, and proper formulations, as seen by the variety of treatment regimens used in all preclinical models. Fluctuating efficiency due to different phases of pathology as well as the dynamic vulnerability of neural cells and brain regions in the human neonatal setting have to be taken into account. Moreover, abnormalities in preterm brain development may differ depending on the inflammatory stimulus as well as the duration of exposure. Furthermore, the relationship between targeted plasma concentrations and clinical efficacy is still unknown. They may vary from those observed in preclinical models of HIE due to a different and more complex underlying pathology [154]. 

In this situation, specific attention must be drawn to defining what constitutes a physiological level of melatonin in preterms in vivo, compared with supraphysiological blood concentrations possibly required for treatment. As a result, a therapeutic threshold associated with neuroprotection and/or neuroregeneration in preterm infants must be examined and compared in greater detail, considering substitutive vs. supra-physiologic regimens. In this context, it appears that better knowledge of melatonin pharmacokinetic/pharmacodynamic (PK/PD) data is required. To perform PK/PD studies on melatonin in preterm infants, the first step would be to define individual physiological melatonin concentrations in a longitudinal manner according to gestational age and stratify based on prenatal/maternal risk factors as well as diseases typically occurring in the premature setting. Close attention to the PK profile in preterm infants is essential, considering gestational age, variation in drug clearance due to immature liver and renal function, as well as interaction with other medications that are routinely administered (e.g., caffeine). Because there are still inconsistencies in plasma concentrations reported thus far [83,145,152,163], which could be due to variability in time of determination, luminosity of environment, and different assays used, bias factors will be minimized by consistency in routine care, blood collection, and performing fully validated analytical methods for quantifying newborns’ low physiological melatonin plasma concentrations. 

**Figure 2:**
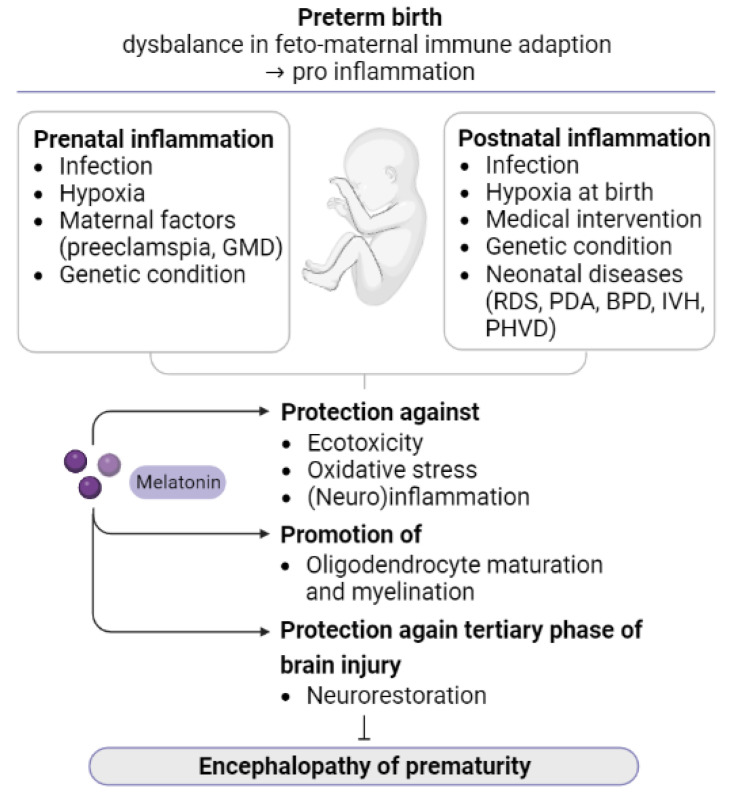
Neuroprotective and neurorestorative effects of melatonin: Prenatal and postnatal risk factors are associated with increased inflammation, which causes injury to the developing brain. Melatonin exerts its neuroprotective effects by reducing excitotoxicity, oxidative stress, and inflammation (acute and chronic), as well as by promoting oligodendroglial maturation and myelination. (Abbreviations: GMD, gestational diabetes mellitus; RDS, respiratory distress syndrome; PDA, patent ductus arteriosus; BPD, bronchopulmonary dysplasia; IVH, intraventricular hemorrhage; PHVD, posthemorrhagic ventricular dilatation).

In summary, melatonin has significant potential as a neuroprotective and neurorestorative agent due to its pleiotropic actions and relatively benign safety profile in both animals and humans (Figure 2). However, some concerns must be resolved before melatonin is introduced into clinical practice. Well-designed research based on translational models is essential to guide the development of evidence-based melatonin use in the preterm setting.

## Figures and Tables

**Table 1:** Effects of melatonin in preterm brain injury (preclinical studies within the last decade).

Species	Study Design	Melatonin Administration	Neuroprotective Effects of Melatonin	Reference
Mice	2× maternal LPS injection at day 15 of pregnancy	once at day 14 of pregnancy 25 mg of melatonin bolus	− preservation of brain structure − prevention of epigenetic changes in the preterm brain − prevention of long-term neurodevelopmental impairment	Domínguez Rubio et al., 2017 [93]
Mice	LPS injection on day 16 of pregnancy	30 min prior to LPS injection 10 mg/kg of melatonin bolus	− mitigation of morphologic changes in postnatal neurons − prevention of adverse neuromotor outcomes	Lee et al., 2019 [94]
sheep	complete umbilical cord occlusion at 130 days gestation	continuous infusion started before and up to 1 h after occlusion 1 mg of melatonin as bolus, followed by a total of 2 mg as infusion	− reduction of neuronal lipid peroxidation − conservation of blood–brain barrier integrity	Yawno et al., 2012 [95]
sheep	in utero complete umbilical cord occlusion in 101 to 104-day gestation sheep	continuous infusion started 15 minutes before to 6 h after occlusion 0.1 mg/kg of melatonin as bolus, followed by 0.6 mg/kg as infusion	− faster recovery of fetal EEG − improved numbers of mature oligodendrocytes − reduced microglial activation in the white matter	Drury et al., 2014 [96]
sheep	complete umbilical cord occlusion in 95 to 98-day gestation sheep	Continuous infusion for 24 h starting at 2 h after HI 0.2 mg of melatonin as bolus, followed by a total of 2.4 mg as infusion	− increase in oligodendrocyte number and improved myelin density within certain white matter areas of the brain − improved neuronal survival within the cortex	Yawno et al., 2017 [97]
Rats	LPS injection into P5 rats	once immediately after LPS injection 20 mg/kg of melatonin	− reduction of neurobehavioural disturbances − reduction of stress-induced brain damage	Wong et al., 2014 [98]
rats	LPS injection into pregnant rats + excitotoxic insult to P4 rats	administration at the same time as LPS application 5 mg/kg of melatonin	− complete reversion of sensitization to second excitotoxic brain damage	Carloni et al., 2016 [99]
rats	LPS injection + left common carotid artery ligation in P2 rats	1 h before LPS injection + once a day for 1 week 15 mg/kg of melatonin, repeated dose	− inhibition of disruption of BBB permeability − improvement in white matter recovery	Hu et al., 2017 [100]
rats	LPS injection in P1 rats	immediately after LPS injection, then once daily until P7 10 mg/kg of melatonin, repeated dose	− amelioration of neurobehavioral disturbances − improvement of axonal hypomyelination in the PWM	Zhou et al., 2021 [101]
rats	LPS injection + ligation of the right carotid artery + hypoxia treatment	1 h before injection + once a day for 7 days 15 mg/kg of melatonin, repeated dose	− attenuation of white matter damage	Qin et al., 2021 [102]
rats	uterine artery occlusion on E18 + LPS injection	+EPO once a day from P1 to P10 20 mg/kg of melatonin, repeated dose	− improved performance in multiple sensorimotor, cognitive, and behavioral domains − attenuation of deficits in social Interaction	Jantzie et al., 2018 [57]
rats	uterine artery occlusion on E18 + LPS injection	+EPO once a day from P15 to P20 20 mg/kg of melatonin, repeated dose	− improvement of gait metrics in rats diagnosed with CP	Jantzie et al., 2022 [56]

Abbreviations: h, hour(s); LPS, lipopolysaccharide; P, postnatal day; E, embryonic day; HI, hypoxia/ischemia; PWM, periventricular white matter.

## Data Availability

No new data were created or analyzed in this study. Data sharing is not applicable to this article.
